# Perceptions of UK medical graduates’ preparedness for practice: A multi-centre qualitative study reflecting the importance of learning on the job

**DOI:** 10.1186/1472-6920-13-34

**Published:** 2013-02-28

**Authors:** Jan C Illing, Gill M Morrow, Charlotte R Rothwell nee Kergon, Bryan C Burford, Beate K Baldauf, Carol L Davies, Ed B Peile, John A Spencer, Neil Johnson, Maggie Allen, Jill Morrison

**Affiliations:** 1 Centre for Medical Education Research, Durham University, Burdon House, Leazes Road, Durham DH1 1TA, UK; 2 Warwick Institute for Employment Research, The University of Warwick, Coventry CV4 7AL, UK; 3 Institute of Clinical Education, Warwick Medical School, The University of Warwick, Coventry CV4 7AL, UK; 4 School of Medical Sciences Education Development, Newcastle University, Framlington Place, Newcastle upon Tyne NE2 4HH, UK; 5 College of Medical, Veterinary and Life Sciences, University of Glasgow, University Avenue, Glasgow G12 8QQ, UK

## Abstract

**Background:**

There is evidence that graduates of different medical schools vary in their preparedness for their first post. In 2003 Goldacre *et al.* reported that over 40% of UK medical graduates did not feel prepared and found large differences between graduates of different schools. A follow-up survey showed that levels of preparedness had increased yet there was still wide variation. This study aimed to examine whether medical graduates from three diverse UK medical schools were prepared for practice.

**Methods:**

This was a qualitative study using a constructivist grounded theory approach. Prospective and cross-sectional data were collected from the three medical schools.

A sample of 60 medical graduates (20 from each school) was targeted. They were interviewed three times: at the end of medical school (n = 65) and after four (n = 55) and 12 months (n = 46) as a Year 1 Foundation Programme doctor. Triangulated data were collected from clinicians via interviews across the three sites (n = 92). In addition three focus groups were conducted with senior clinicians who assess learning portfolios. The focus was on identifying areas of preparedness for practice and any areas of lack of preparedness.

**Results:**

Although selected for being diverse, we did not find substantial differences between the schools. The same themes were identified at each site. Junior doctors felt prepared in terms of communication skills, clinical and practical skills and team working. They felt less prepared for areas of practice that are based on experiential learning in clinical practice: ward work, being on call, management of acute clinical situations, prescribing, clinical prioritisation and time management and dealing with paperwork.

**Conclusions:**

Our data highlighted the importance of students learning on the job, having a role in the team in supervised practice to enable them to learn about the duties and responsibilities of a new doctor in advance of starting work.

## Background

One of the fundamental aims of all medical schools is to ensure medical graduates are prepared to start work safely as junior doctors. While diversity of curriculum approaches in medical schools is encouraged, each medical school in the UK must ensure that the outcomes specified in the General Medical Council’s (GMC) *Tomorrow’s Doctors* are attained by students on graduation [1]. However, there is evidence that graduates of different medical schools vary in their preparedness for their first post. In 2003 Goldacre *et al.*[2] reported that over 40% of UK medical graduates did not feel prepared and found large differences between graduates of different schools. A follow-up survey showed that levels of preparedness had increased yet there was still wide variation [3]. Lack of preparedness has also been recognised internationally with reports of students being less prepared than they should be for starting practice [4-8]. In addition, the longer-term relationship between medical school and career progression is illustrated by findings that performance in certain postgraduate examinations can vary with place of graduation [9].

Several studies have compared different types of curricula; in particular, graduates of traditional curricula have been compared with those of problem-based learning (PBL) courses [10-13]. PBL involves more student-led, small group work, and less didactic teaching than ‘traditional’ approaches. Findings indicated that PBL programmes may be more effective at preparing trainees for some aspects of their first medical jobs, such as dealing with complex clinical problems, working in a team, being aware of limitations and knowing when to ask a senior for help (behaviour which has been identified as a primary indicator of a trainee’s competence [14]). However, a recent systematic review did not provide conclusive evidence of any distinctive effect of PBL [15].

Another change in recent years is the expansion in graduate-entry medical education in the UK. Extensive literature looking at the impact of these programmes is yet to emerge, but two early studies reported either no or few differences between graduate and non-graduate entrants [16,17]. The differences were more to do with quality of life issues such as time available for family, working hours and living conditions than with clinical differences.

Studies that have explored preparedness for practice have identified the change in status from student to doctor as challenging and stressful [18-20]. There is evidence that junior doctors are well prepared for communication skills and working in teams[21-26], but studies have reported junior doctors experienced a lack of confidence in clinical and practical skills (although this improved after one month in post) [27] and a lack of competence in prescribing [3,21,26-28]. One study capturing both the views of junior doctors and consultants reported that junior doctors were least prepared for prescribing, treatment, decision-making and emergencies [26]. Recently published work found consultants and specialist registrars reported that trainees were not prepared for starting work particularly with regard to clinical and practical skills and the more challenging aspects of communication [29]. In a report for the GMC on the implementation of *The New Doctor* the authors stated that educational supervisors and managers reported curriculum changes had been detrimental and the curriculum did not prepare trainees well enough [30].

This problem is not unique to the UK. Langdale *et al.*[4] comment that US medical schools consider their curricula successful if students pass the national examinations, but highlight that physicians need more than the requirements of the national exam to function with even a modest degree of independence. They also report on the gap between medical school objectives and residency-specific expectations. Residency directors report having to spend up to six months confirming competencies.

The GMC has recently revised *Tomorrow’s Doctors*[31]. The study reported here, examining the preparedness of graduates from three different medical schools, contributed to this process. Currently the literature indicates that graduates’ preparedness for practice varies with medical school but we do not understand why. It is possible that the curriculum prepares medical graduates differently [32]. The aim of this study was to explore whether UK medical graduates were prepared for medical practice and understand more about why they were or were not prepared.

## Methods

### Design

The study compared the perceptions and experiences of new graduates (followed up into their first year of practice), their tutors, educational supervisors and members of clinical teams. Three different types of medical school were compared:

1. Medical School 1, which has an integrated systems-based curriculum, mainly undergraduate entry.

2. Medical School 2, which is wholly graduate entry (at the time of the study restricted to bioscience graduates).

3. Medical School 3, which is based on a PBL curriculum, with mainly undergraduate entry.

Following the findings of earlier studies [2,3] it was anticipated that differences would emerge due to the schools’ diversity.

### Conceptual framework

This study was conducted using a grounded theory approach; as such typically “a researcher does not begin a project with a preconceived theory in mind” [33],p12_,_ however a theory may emerge from the data and other pre-existing theory may be found to be relevant to the findings. This approach is called abductive reasoning which has been explained as: studying individual cases inductively and discerning a finding and then asking how theory could account for it [34], p103.

Strauss and Corbin [33],p15 explain theory as “A set of well-developed concepts related through statements of relationship, which together constitute an integrated framework that can be used to explain or predict phenomena”. Constructivist grounded theory differs in that more emphasis is placed on *understanding* rather than explanation [34],p126. Constructivist grounded theory “places priority on the phenomena of study and sees both data and analysis as created from shared experiences and relationships with participants and other sources of data …and also acknowledges that the resulting theory is an interpretation” [34],p130.

The work of Lave and Wenger [35] is referred to here, by way of introduction, to theory that was found to be relevant to the findings of this study. Their work highlights the importance of learning in the workplace (based on their study of five different cases of apprenticeship). They discuss the idea of the workplace being more focused on learning rather than teaching [35], p92. The community who practice what is to be learned “creates the potential ‘curriculum’ in the broadest sense - that which may be learned by newcomers with legitimate peripheral access”. This idea of the apprentice learning by working on the job and by observing and participating in work of the community has relevance to how doctors may learn in the clinical setting. In the past medical training did involve final year medical students undertaking more clinical practice in the workplace by taking on locum posts and having a role in ‘the firm’ (team) where they were allocated patients to be responsible for. With the increase of concern about and focus on patient safety these ways of learning are today significantly diminished and medical students learn and rehearse their skills away from real patients using mannequins, standard patients and simulation. The benefits of these techniques are without question, particularly with regard to skill acquisition [36,37], however the filtering out of reality may leave the learner less prepared for the ‘crowdedness’ [38] of the real context. Eraut has separately highlighted the importance of informal learning from placements and gaining tacit knowledge [39]. These theories have underpinned the findings of this study and support a deeper understanding of the issues explored.

### Participants

UK medical graduates proceed to a two year Foundation Programme in which they generally complete six four-month placements in different clinical specialties. In Foundation Year 1 (F1) they work under supervision, and have provisional registration with the GMC. By the end of the first year they must have demonstrated sufficient competence for full registration, and are expected to take more responsibility in Foundation Year 2 (F2).

From this point on graduate refers to doctors who have graduated from medical school. The graduate sample consisted of 65 doctors. We intended to recruit 20 who had recently qualified from each medical school, but Medical Schools 2 and 1 over recruited (by 4 and 1 respectively). The sample was interviewed three times: at the end of medical school (n = 65) and after four (n = 55) and 12 months as a Foundation doctor (n = 46).

The graduate sample was selected purposively to achieve maximum variation of participants, firstly to ensure variation in ability (using the academic Medical Training Application System (MTAS), a national ranking system for junior doctors), with at least five students being sampled from each MTAS quartile. Secondly the sample was also selected to ensure representation in terms of gender, age, ethnicity and disability.

Three groups were identified as having insight into the transition of medical graduates into Foundation Year 1: undergraduate tutors, who oversee students in their final year and so are aware of their level as they approach the end of their undergraduate programme; F1s’ educational supervisors, who are responsible for identifying learning goals and development aims with trainees, and so should be aware of their strengths and weaknesses at the beginning of the year; and key managers with programme-level responsibility for groups of trainees. In practice the groups overlap, with some undergraduate tutors and key managers also having supervisory contact with F1s, as educational or clinical supervisors, but for recruitment some distinction was made, with individuals being recruited with a primary focus on one role or another.

Members of these populations were identified by the research team in consultation with key medical school and Foundation School faculty, and individuals were invited to take part in a telephone interview by letter with an enclosed information sheet. The majority of those approached agreed to take part, and the interview was conducted at their convenience, recorded with consent, and transcribed. Interviews typically lasted 20 minutes.

Triangulated data were collected from interviews with 92 clinicians over the three sites (33, 27 and 32 at Medical School 1, 2 and 3 respectively), including 28 undergraduate tutors, 29 educational supervisors, 17 health managers and 18 members of the clinical teams who worked with these new doctors. These interviews took place between 4 and 6 months after the graduates started work as a doctor.

In addition, a focus group was held at each site with a panel of portfolio assessors to explore strengths and weaknesses in training and preparedness for practice as demonstrated in learning portfolios submitted by the 2006–2007 cohort of F1s in July 2007.

### Procedure

Before interviews took place with the graduate sample, six focus groups (two at each site) were held with volunteer final year medical students and newly qualified doctors in the first and second year of the Foundation Programme, in order to guide the development of the initial interview schedule.

Graduate sample interview questions were open-ended and semi-structured. The first interview (conducted face-to-face) started with questions on the areas for which the interviewee felt prepared or not prepared. Themes that emerged in early interviews were explored further in subsequent interviews, conducted by telephone. Interviews took 20–60 minutes and were conducted by eight researchers. The Medical School 1 interviewers (JCI, GMM, CRR and BCB) collected data at all sites supporting the other teams and were not involved in the delivery of teaching. The other interviewers were social science research staff. None of the interviewers were medically qualified. Regular meetings were held between researchers to ensure consistency of approach.

Interviews with clinicians (the triangulating sample) followed the same format using similar questions to the interviews with the graduate sample. In addition, three focus groups (one per site) were conducted with senior clinicians who assess learning portfolios, to determine areas of preparedness, and lack of preparedness, for practice following assessment of the portfolios.

In total, over 250 qualitative interviews were conducted.

### Interview schedule

The interview schedule for the triangulating interviews was developed concurrently with the primary sample follow-up schedule, and focused on the same broad areas of preparedness, with some targeted probes derived from the initial primary sample interviews. Interview schedules varied slightly with the primary role in which respondents were contacted, but had the same basic structure:

•Role with undergraduates/F1s

•Perceived preparedness of graduates before starting F1

•Problems and strengths in performance during the first four months of F1

•How typical were the current F1s compared to previous cohorts

•Recommended changes to undergraduate programme

### Analysis

The analysis was conducted using constructivist grounded theory [34], an iterative approach which aims to develop theory from data. This perspective maintains that research findings are created from the relationship between the researcher and the object of study. The researcher gathers data and looks for new meaning based on relative consensus within the data [33,34,40].

Regular meetings were held with all interviewers at each stage of analysis. Transcripts of interviews were read and coded separately and discussed at each site and at interviewer meetings to agree on themes and sub-themes and to ensure theoretical saturation (the point when no new themes or properties are emerging, typically reached between 10 and 15 interviews). Themes were identified at each medical school and explored and discussed to determine if the theme applied to other medical schools. A coding frame was agreed at these meetings to enable direct comparison of data coming from each medical school and data was coded using NVivo software.

Data collection and analysis followed theoretical sampling which “involves starting with the data, constructing tentative ideas about the data, and then examining these ideas through further empirical inquiry” [34],p102 .“Quite possibly you might ask participants further questions or inquire about experiences that you had not covered before” [34],p103 .

Analysis of the first interviews with the graduate sample identified a number of themes reflecting graduate perceptions of preparedness or lack of preparedness for practice. These concerns and others from the triangulating sources fed directly into follow-up interview schedules which explored whether or not they were realised in practice and were relevant for all respondents. Subsequent interview schedules were also agreed at researcher meetings and developed to follow up issues that emerged from the data to fully understand them and identify any change over time.

Later interviews with the graduate sample and interviews with educational supervisors, undergraduate tutors, health managers and clinicians who worked with the new doctors were analysed using these themes, which were extended and developed where appropriate to fully understand and explain the data. Further analysis refined the themes to help us understand the circumstances under which certain areas of practice were a concern, and what helped reduce this.

The second follow-up interview was used to gain respondent validation of findings. Respondents were sent the executive summary of an initial report to the GMC [41] and asked about their views on the findings. Respondents agreed with the findings although one recommendation was modified following respondent feedback.

## Results

### Demographics of the sample

In total from the three Medical Schools there were 25 male and 40 female graduates in the study (11,13 and 16 females from Medical School 1,2 and 3 respectively). Nine were identified as being from an ethnic minority group (2,2,5) and two classified themselves as disabled (1,0,1). There were nine graduates aged 30 or over (3, 1, 5). The profile of the sample broadly reflects the demographics of the cohorts in which there are more females, particularly at Medical School 3 (59%, 54%, 69%) and low percentages of ethnic minorities, mature students and students who are classified as disabled.

There were no indications of any variation in responses associated with MTAS quartile or demographic variables.

### Focus of the paper

This paper focuses on the two main themes: (1) areas of preparedness and (2) lack of preparedness; those interested in the full dataset are referred to the initial full report [41] or for a summary of findings including third interviews see the final report to the GMC [42]. The quotes presented refer to the medical graduate interviews at first interview, after 4 months in post or after 12 months in post. The quartile refers to the MTAS score. The other quotes relate to other members of the clinical team.

The results highlighted the relevance of the work of Lave and Wenger to this study [35]. The themes presented below have been explained by reference to their work.

The analyses of the data led to the generation of eleven major categories (listed below). The process of grounded theory aims to identify a central or core category that links categories into a larger theoretical scheme [33]. “A central category has analytical power. What gives it that power is its ability to pull the other categories together to form an explanatory whole” [33],p146-147. The core category that emerged from the data (there were no specific questions addressing it) was on-the-job learning. It became evident from the analysis that this core category was consistently present linking data to preparedness for practice (when present) and lack of preparedness (when absent). Preparedness for different areas of practice varied with the extent and quality of the exposure to clinical practice the graduates had received as medical students. The grounded theory that emerged from the data suggested that in addition to learning on the job, passive exposure or observation was not sufficient for preparedness. Final year students needed to have a role in the team and be supervised doing appropriate clinical tasks prior to starting work.

Categories emerging from the qualitative data analysis:

•Transition –‘Becoming a doctor’

•Factors that impact on preparedness

•The role of the F1 (intern) and other team members

•Managing the duties of a doctor

•Knowledge

•Clinical and practical skills

•Prescribing

•Communication

•Use of a learning portfolio

•Identifying learning needs

•Improvements to training

### Differences due to medical school

Although selected for being diverse, we did not find substantial differences between the graduates of the three medical schools regarding preparedness. The same themes were present at each site. However, some minor differences were identified. For example, in Medical School 2 there was a suggestion that the maturity implicit in having already completed a first degree helped in situations requiring more complex communication skills. Some respondents felt that Medical School 3 students, having followed a PBL course, were more assertive in their approach to asking for help.

### Areas of preparedness

#### Foundation doctor role and team working

Before starting work students reported they did not have a role in the clinical team and were frequently observers. As an F1 they were expected to participate. In relation to the work of Lave and Wenger this illustrates their transition from having a right to be there (legitimate) in an observational and non-participative role. The quotes below highlight a movement away from being an observer to being a participant in the activities of the community of practice.

*“They always say that students are part of a team but it’s not true.”* (Medical School 3, PS11, first interview, quartile 1)

*“Suddenly I have to be the one to make the decision and I feel like I have a role in the team this time around, instead of just being an observer.”* (Medical School 1, PS143, 4 month follow-up, quartile 2)

In addition to this, opportunities to do more than observe were identifiable as either ‘external’ to the student, such as being placed in a particularly supportive team, or ‘internal’ to the student, such as having a more assertive personality and asking to perform many procedures.

*“[it depended on] which doctor … was there and whether he was keen on letting me do it, or whether he or she was busy”.* (Medical School 1, PS182, first interview, quartile 2)

*“You inevitably get some people who are a bit shy and, you know, find it difficult to put themselves forward and to some extent they have to learn to assert a little bit more authority”.* (Medical School 1, educational supervisor 3)

Generally, the graduates of each of the three medical schools reported that they had some knowledge of the F1 role before starting work. After shadowing and four months of working as an F1 doctor, there was an increase in clarity about their role and how they related to the multi-disciplinary team. Working as part of a team was generally a positive experience; however, some teams provided more guidance and support than others.

The clinicians’ perceptions indicated that the junior doctors were prepared for some aspects of their role but not all, commenting in particular that in the past junior doctors had usually started work with more on-the-job experience having often acted as locums in their final year. Clinicians also mentioned the need for graduates to become part of the team, having a role in the team rather than passively observing. Lave and Wenger have identified that with increasing skill the apprentice gradually moves from newcomer on the periphery of activity towards the centre with increasing skill and expertise. The clinicians here were highlighting a need for this transition to take place; in order for the newcomers to take on more responsibility they also need to take on a role in the team and be responsible for certain tasks.

*“Not passively observing…but part of the ward round and part of the team.”* (Medical School 1, undergraduate tutor 2)

### Communication skills

The main area of preparedness, which was strong in all datasets from all schools, was communication skills. This included communication both with patients and with staff. However, there was evidence from both the graduate sample and the clinicians’ data that more complex communication skills, such as breaking bad news, needed to be developed and that this was best achieved in the workplace.

*“Communication skills I felt fairly prepared for.”* (Medical School 1, PS209, first interview, quartile 1)

Some doctors reported that dealing with bad news was still challenging at the end of their first year as a doctor. Additional challenges were raised at the end of their first year of work – these were dealing with poor communication and conflict between staff members which had not emerged earlier.

“*It’s not always necessarily that you’re breaking bad news, I think you just need to see kind of more examples of people, explaining things, dealing with difficult relatives, that sort of thing*.” (Medical School 1, PS9, 12 month follow-up, quartile 4)

Communication skills can be practised as a peripheral participant, but expert communication such as is required when breaking bad news requires the participant to have moved to the centre of the community. According to Lave and Wenger it would follow that expert skills would be difficult to acquire as a newcomer on the periphery of the community.

### Clinical and practical skills

Generally clinical and practical skills, including history taking and basic procedures such as venepuncture, were areas of preparedness, although graduates from each school reported knowing students who had been signed off as competent following training on mannequins only. Before starting work some graduates expressed concerns about a lack of practice in performing some procedures such as catheterisation, however at first follow-up they reported that they quickly ‘got up to speed’ and in fact had, in retrospect, been better prepared than expected. Perceptions from the clinicians’ data were that while junior doctors developed skills quickly, they would be better prepared if they had spent more time in clinical practice with patients and with clinicians.

*“Some of them have got quite good practically at taking history of patients, listing concerns and things like that.”* (Medical School 1, Health Manager 6)

The view of the clinicians highlights the importance they attribute to workplace learning. They also indicated a need to move from observer to participant, increasing their skills and moving towards the centre of activity.

### Lack of preparedness

#### Working in the hospital on the wards, managing paper work and working on call

The central activity to which all new doctors must adjust when they start work is the practical side of working on a ward. Some of this requires knowledge about local logistics – for example, location of the forms for ordering tests and the correct procedures for ordering an x-ray.

In terms of the responsibility of being a doctor to whom other professions look for medical opinion, graduates recognised that there are practical issues in adapting to the day-to-day environment of the ward for which a purely educational environment would be unable to prepare them. There was a feeling from all schools that more extensive experience on wards as an undergraduate would have provided them with the set of skills necessary for starting work as a junior doctor.

*“I don’t feel that medical school prepares you at all for any sort of ward work in any sort of way really.”* (Medical School 2, PS3, 4 month follow-up, quartile 1)

The contextual elements of the workplace are not easily provided or replicated in a simulated environment. This requires real exposure to the clinical environment and underlines the important skills that need to be learned in the workplace. Lave and Wenger argue that an extended period in practice provides the learners with opportunities to make the culture of practice theirs [35],p95.

A specific subset of ward skills is the appropriate use of the various forms and other paperwork involved in day-to-day ward practice. This routine work is important yet it is not usually a formal part of undergraduate training and tends to get picked up on the job. Awareness that different hospitals may have different systems caused concern to some.

*“You… presume if you write urgent on it, it will happen urgently and then it doesn’t.”* (Medical School 1, PS93, 4 month follow-up, quartile 3)

Being on call, meaning the person who is ready to respond, the doctor on duty, was a common area of concern. This was an area that students seemingly had little exposure to. Concerns were particularly focused on having to make decisions on their own, especially at night when less immediate support was available. While senior support was always available, it can take longer to arrive at night, and there was an added consideration that they may be disturbing the senior unnecessarily. At the end of the first year, the majority of junior doctors still reported that the particularly stressful periods included being on call and working nights although this was now more manageable.

*“I think starting on nights was really tough.”* (Medical School 1, PS18, 4 month follow-up, quartile 4)

*“In hospital they are very supervised, apart from on nights…that’s the fear, where they are most exposed.”* (Medical School 3, educational supervisor 5)

Working in situations that demand more responsibility from the new doctor was a concern to them. The discomfort can be explained by their having to work more centrally (away from the peripheral role) but when a senior is not present.

### Management of acutely sick patients

There were concerns from graduates of each medical school about making clinical decisions and patient management. Particular concerns were expressed about taking immediate steps with acutely ill patients. Being the first doctor to deal with a sick patient remained a concern even at the end of the F1 year.

*“I’ve had difficulty with being in the acute situation…being the first person to initiate basic management for that patient and recognising what’s wrong.”* (Medical School 1, PS26, 4 month follow-up, quartile 4)

*“The hardest thing for them is the acute on calls. I think they struggle with assessing truly sick patients.”* (Medical School 1, educational supervisor 14)

Much of the concern about working on nights was focused on responding to acutely sick patients. The junior doctors are expected to call for help appropriately but are also aware that there will be situations that require them to act before help arrives. This places the junior doctor in the central role when as a learner they are more comfortable being more peripheral and learning in the presence of experts. The Lave and Wenger [35]*central-peripheral* positions of team members highlights a disjunct in the expectations from the service during the day and night when the junior doctors move from a peripheral learning position to a central position of first response to acutely sick patients.

### Prescribing

Prescribing was perceived to be the weakest area of practice, confirmed by data from all three medical schools and from all sets of data. This weakness generally covered the breadth of knowledge and skills related to prescribing, encompassing both pharmacological knowledge and the practicalities of prescribing. Frequent reference was made to under-preparedness with regard to the common drugs and doses which they would use in practice.

In addition, it was perceived that the main occurrence of error was in relation to prescribing. Due to the possibility for such errors to cause harm, this constitutes a significant potential risk. However, the risk was reduced by ward pharmacists, who were found to be consistently helpful and effective in noticing mistakes.

*“I think you feel just a little bit silly when you don’t know common doses.”* (Medical School 1, PS143, 4 month follow-up, quartile 2)

*“There is one area where they aren’t prepared and that’s prescribing.”* (Medical School 2, educational supervisor 4)

The lack of preparedness for prescribing is understandable given this is a high order activity. The junior doctors were working somewhere between a peripheral and central role in that they were expected to manage the task of prescribing but many errors were picked up after the event by pharmacists and nurses. Prescribing is an activity that is difficult to teach and learn away from the clinical setting and is best learned on the job. Again the Lave and Wenger model highlights a conflict between novice and expert, where the novice junior doctor is expected to perform a complex skill.

### Prioritising patients and time management

Most junior doctors reported they were not prepared for prioritising their workload or managing time. While in part this simply reflected their personal time management, there were also worries about clinical prioritisation – for example, knowing which patient to see first. At the end of the year most reported that their time management had improved, although several still talked about a heavy workload.

*“Initially I might struggle to prioritise my jobs.”* (Medical School 1, PS14, 4 month follow-up, quartile 1)

*“The big thing that has come through with quite a few of my trainees is time management…the other problem is that… it takes a long time to learn how to prioritise.”* (Medical School 1, educational supervisor 3)

The final year of training left the students so peripheral to practice that they were having to consider time management and the prioritisation of patients and tasks for the first time when starting work. The service expected certain tasks would be achieved within a certain time period and it was only after working in post for 12 months that improvements were reported. Managing workload and prioritising patients are processes that are not easily simulated away from the workplace and have more meaning when they are combined with participation in a community of practice in the real workplace.

### Shadowing

The importance of developing expertise in the workplace was reinforced by data collected from the clinicians who worked with the new junior doctors. Suggestions included having more exposure to clinical practice and having a longer shadowing period before starting work to gain more understanding of the day-to-day life of a junior doctor and to become more integrated into the team.

“*Shadowing means just watching…what I mean is actually taking part in but not being solely responsible*”. (Medical School 1, educational supervisor 19)

*“I think an attachment as a final year medical student is two months of work shadowing that sort of thing, where they actually get involved with the ward during that time”.* (Medical School 3, undergraduate tutor 9)

The clinicians stated that to be more prepared for starting work the final year students need to take on more responsibility towards the end of their training in supervised clinical practice with a clear role in the team. Shadowing the post before starting work is a legitimate, but slightly less peripheral role than being a student. It also provides a safety net for those who have been hesitant to become more participatory, enabling them to engage more with the new role which is ahead of them.

## Discussion

Previous studies have reported that new doctors’ preparedness varied with medical school attended but did not explain why students of one medical school were better prepared than those of another school. Our findings indicate that the factors that need to be present to support preparedness for practice are opportunities for learning on the job and having a role that enables engagement in supervised clinical practice. Previous literature did not identify that the amount of learning on the job influenced preparedness.

As before, communication has been identified as a strong area of perceived preparedness [18-20,25,26,30]. However, our data suggests that these new doctors quickly ‘got out of their depth’ when dealing with the more complex communications such as dealing with patients and relatives following bad news, a finding also reported elsewhere [10,29]. Some clinicians felt that no amount of rehearsal could fully prepare graduates for the real thing, but having greater exposure to these situations, including being exposed to complex communications as a result of being on the wards more as students, would have given medical graduates more preparation in the past. There was a limit to the extent to which certain aspects of work could be learned or rehearsed in a classroom setting and then transferred to the clinical setting. These included topics identified under the qualitative theme ‘managing the duties of a doctor’, such as working on call, acute care and prioritising work. It was these areas, together with prescribing, that were identified as the areas in which F1s were less prepared and it was recognised that these skills are best learned on the job, indicating that in terms of preparation for practice other forms of learning are not a substitute for real ward experiences. These areas of weakness have also been reported elsewhere [28,29,43].

The data analyses led to the identification of one core category ‘on-the-job learning’ that pulled the other categories together and accounted for variation within categories. It highlighted the importance of exposure to real practice, in the real context with all the complexity and crowdedness of the clinical environment. There has been an assumption that students can learn effectively away from the clinical environment [44] and can be signed off as competent in alternative settings such as a classroom or a simulated environment. Our findings stress that learning outside the clinical environment may not be equivalent to real clinical experience; there are routines, procedures and knowledge that it is felt can only be imparted and experienced in the clinical environment. Our findings indicate that better preparedness for practice is achieved from more exposure to clinical practice that involves supervised practice. Supporting evidence for this is found elsewhere: for example, students from those medical schools that have a full year of practice following final exams self-rated themselves highest on preparedness [3,45].

The current generation of junior doctors in the UK generally start work with less on-the-job experience than earlier generations. There are several reasons for this. Most obvious is the demise of the student ‘locum’ post where undergraduates in the past would work as junior doctors. Changes in practice and concerns over patient safety put an end to this. The Foundation Programme is more closely supervised than earlier posts. There are also changes in the in-patient profile, with more severely ill patients with shorter hospital stays, giving students less opportunity to see patients as the patients tend to be receiving “management” e.g. x-rays, or surgery and so are less available for students to examine. The organisation of the clinical workforce has changed. Teams have changed from the stability of ‘firms’ to transient teams that are constantly changing due to shift work, shorter placements and in response to the dispersal of patients across many wards. The increase in student numbers has also increased the competition for access to learning opportunities with patients. A consequence of these changes is that the graduates in our study reported an absence of a role during clinical placements. They have been outsiders, in the role of passive observers. Dornan [46] argues that apprenticeship has come under severe strain. However apprenticeship still enables the learning of tacit knowledge which is best achieved through modelling in practice.

This paper has attempted to relate the findings of the study to the work of Lave and Wenger on communities of practice with emphasis on apprenticeship learning and working within a community of practice [35,47]. The apprenticeship model of legitimate but peripheral participation highlights problems when final year students continue to observe rather than be supported in supervised practice (thus hindering their preparedness for practice as a new doctor).

Lave and Wenger [35] argue that learning must be understood with respect to practice as a whole. Above we have suggested why students do not get access to practice as a whole, with the main driver for change being a concern with patient safety. This focus on patient safety has led to a move away from learning on the job and into other contexts, such as simulation. In the final year, students need to be more prepared for practice by taking on more legitimate tasks in preparation for the workplace. The novice student is initially ‘peripheral’ to the team but with increased competence, particularly in the final year, the student needs to participate in practice and acquire skills to support the more independent status expected of an F1 doctor. This would involve the final year student having a clear role and becoming part of the team.

Wenger [47], p74 states that “being included in what matters is a requirement for being engaged in a community’s practice”. Wenger argues that both participation and reification are required to negotiate meaning. Wenger [47],p55 makes a distinction between participation and reification; (referring to the processes and objects that have meaning within the community of practice [47],p59).This helps us to understand that participation is not enough; there is a need to engage with processes. The junior doctors found processes such as managing paperwork, prescribing and prioritising patients were as challenging as participation, which at times involved being the first to respond to an acute scenario and deciding what actions to start as well as who to call. Wenger [47], p164 highlights that participation and non-participation also contribute to the formation of professional identity, stating that non-participation also serves to define identity and role. Certain tasks and responsibilities were expected of the junior doctors, but at times the expectations became much broader when no senior doctors were able to respond quickly.

Wenger describes three dimensions of practice as the property of a community: mutual engagement, joint enterprise and a shared repertoire. Mutual engagement requires “not only our competence but also the competence of others” [47], p76, this is particularly meaningful in the context of starting work as a junior doctor. Joint enterprise is the negotiation of a shared activity and a shared repertoire includes “routines, words, tools, ways of doing things…” [47], p83. It is clear that accessing the joint enterprise and shared repertoire is challenging when less training is provided on the job.

We know from the interviews that students were signed off as competent to perform certain tasks but these tasks were frequently in simulated contexts. The competency literature tends to assume that competencies are generalisable but Eraut [39] comments that there is little evidence to support this. Eraut highlights the importance of identifying the domain in which an individual is deemed to be competent by considering context, conditions and situations.

The transition from newcomer to expert is illustrated in the five stage model by Dreyfus and Dreyfus [48] in which the novice arrives with little situational judgement and, with increasing exposure and experience, gains tacit knowledge. Competence is reached at level three when the learner is able to cope with crowded busy contexts again reflecting the importance of learning in practice. Eraut [39] stressed that newly qualified professionals survive by learning how to reduce the cognitive load by learning how to prioritise and routinise their work. This process then frees up more time for thinking and to interact with others. Moreover Eraut stresses the importance of working alongside others in practice. This provides the opportunity to gain different types of knowledge including tacit knowledge, about the workplace. Lastly Eraut also [44] suggested that confidence to proactively seek learning opportunities is an important factor in workplace learning. Our data highlighted the importance of students having a role in the team in supervised practice to enable them to learn about the duties and responsibilities of a new doctor in advance of starting work. Similarly Cantillon and MacDermott [49] argue that real practice and responsibility drives learning.

Our study compared three medical schools, selected because they were diverse. We expected to find differences in preparedness but did not. With similar amounts of on-the-job training (28–32 weeks) but different curriculum approaches and student intake, students’ preparedness/lack of preparedness was the same – the missing element being participatory learning opportunities in the workplace.

### Limitations

This paper has focused on perceptions of preparedness to start work rather than using an external objective measure of preparedness. However, perceptions are important as they guide and impact on performance, confidence and ability to do the job. In this study, the perceptions of the trainees were very similar to those of experienced clinicians.

There were slight differences in sampling due to the time constraints for recruitment, with Medical School 3 forced to take a more opportunistic approach. Nonetheless, the Medical School 3 sample did represent a demographic cross-section and a range of MTAS scores.

## Conclusion

Data from trainees, and from those working with them, indicated a need for more ‘on-the-job’ training prior to starting work. Recent work by others supports our important finding [22,28,43]. Learning how wards operate, about the work of a junior doctor and how to prioritise work are all best learned on the job. Graduates today have less time in clinical practice, with direct patient contact and involvement in day-to-day ward business, than was the case for earlier generations of medical students; this balance needs redressing to support better preparedness for practice. The work of Lave and Wenger [35], Wenger [47] and Eraut [39] has provided a conceptual lens which has helped highlight some of the problems medical graduates experience when making the transition to the workplace.

### Implications

Our findings indicate a need for increased on-the-job training. We think this is best achieved by improving the structure of medical school placements to ensure greater consistency and validity in student experience. Students need to be given a role within the team to increase participation and enable more supervised clinical practice. Preparedness would be enhanced by ensuring new graduates shadow their first post in addition to gaining more ‘in-depth’ experience. This may then mitigate against the lack of preparedness for managing patients, including acute scenarios and for managing the hospital workload including the paperwork. Finally, particular weaknesses in prescribing need to be addressed by supporting the development of applied prescribing in the clinical setting - a finding that has been supported by more recent research [28].

The findings of this study have informed the latest edition of the GMC’s *Tomorrow’s Doctors*[31]. One major change has been the introduction of the *student assistantship* into medical students’ final clinical placements. The student assistantship will enable students to have roles in the teams and be engaged in supervised practice in advance of starting work as registered doctors thus enhancing opportunities to be more prepared for practice.

### Further research

The core finding that preparedness is directly related to experiential learning should be explored further by comparison with other medical schools. Ideally, the student assistantships should be evaluated to identify whether preparedness for practice has improved. A detailed study on prescribing skills should be undertaken, particularly with reference to interventions to improve prescribing skills, and a study exploring the tensions of being a novice doctor while meeting the needs of covering 24/7 health service should be considered.

### Ethical review

The study received ethical approval from the NHS National Research Ethics Service, Cambridgeshire 2 Ethics Committee.

## Competing interests

The authors declare that they have no competing interests.

## Authors’ contributions

JCI, GMM, CRR, BCB, BKB, CLD, EBP, JAS, NJ, MA and JM designed the study. JCI, GMM, CRR, BCB, BKB, CLD and JM conducted the analysis. JCI, GMM, CRR and BCB drafted the manuscript. All authors read and approved the final manuscript.

## Pre-publication history

The pre-publication history for this paper can be accessed here:

http://www.biomedcentral.com/1472-6920/13/34/prepub
